# Integrated Dynamic Modeling and Ground Test Validation for Spacecraft Micro-Vibration Suppression Considering Disturbance, Isolation, and Pointing Control

**DOI:** 10.3390/s26113534

**Published:** 2026-06-03

**Authors:** Hua Wang, Han Yan, Lei Tian, Xu Zang, Yingqing Zu

**Affiliations:** 1 Department of Aeronautics and Astronautics, College of Intelligent Robotics and Advanced Manufacturing, Fudan University, Shanghai 200433, China; 2 Aerospace System Engineering Shanghai, Shanghai 201109, China; 3 Institute of Tracking and Telecommunication Technology, Beijing 100080, China

**Keywords:** micro-vibration, integrated model, full-link analysis, ground experiment

## Abstract

On-orbit micro-vibration has emerged as a critical constraint impairing the imaging performance and ultra-high pointing accuracy of space optical payloads. Most existing investigations separately concentrate on disturbance modeling, vibration isolation design, or line-of-sight (LOS) stabilization, leaving the full-link integrated dynamic modeling and analysis severely insufficient. To address this gap, this paper proposes an integrated dynamic modeling methodology for spacecraft equipped with optical payloads, which synergizes disturbance identification, finite element modeling, model order reduction, hybrid active–passive vibration isolation mechanism control, and fast steering mirror (FSM) regulation. The experimental and simulation results demonstrate that the root mean square (RMS) acceleration induced by flywheels and pumps at the mounting interface of the vibration isolation mechanism approximates 4.50 mg. Specifically, the passive vibration isolation scheme attains an attenuation of −16 dB, while the hybrid active–passive strategy achieves a remarkable −30 dB attenuation. Moreover, flywheels generate lower acceleration amplitude but more severe LOS jitter, owing to their time-varying disturbance characteristics and dispersed frequency energy distribution. Additionally, a full-spacecraft micro-vibration ground test incorporating horizontal gravity unloading via suspension is implemented to validate the model. The model-calculated acceleration and pointing angle exhibit excellent consistency with the experimental data, with the relative acceleration error below 7% and the angular error less than 9%. The proposed integrated dynamic model enables accurate prediction of micro-vibration transmission and suppression performance, laying a dependable theoretical foundation for design optimization of high-precision spacecraft systems.

## 1. Introduction

On-orbit micro-vibration has been universally identified as the dominant constraint that severely degrades the imaging performance, pointing precision, and line-of-sight (LOS) stability of high-resolution space optical payloads, including Earth-observation cameras, laser communication terminals, astronomical telescopes, and gravitational-wave detection payloads [1,2]. With the rapid evolution of space remote sensing and optical engineering [3], modern optical payloads are advancing toward ultra-high resolution, ultra-lightweight structures, sub-arcsecond pointing accuracy, and long-term on-orbit stability [1,4]. For example, for payloads with resolution better than 0.3 m, a jitter of 0.1 μrad causes image distortion exceeding one pixel, directly failing high-precision imaging requirements [2]. Thus, micro-vibration suppression has become a core technical bottleneck in the design of advanced high-precision spacecraft.

On-orbit micro-vibration disturbances of spacecraft can be divided into internal disturbances and external disturbances. Internal disturbances are mainly generated by rotating and moving components, including flywheels, control moment gyroscopes, solar array drive assemblies, and antenna drive mechanisms. External disturbances include thermal shock, solar radiation pressure, and micro-meteoroid impacts, among which thermal shock caused by the alternation of sunlight and shadow in orbit is a typical disturbance [5,6,7]. The temperature gradient between the sunny and shadow sides of the spacecraft can reach 270–300 °C, resulting in thermal deformation of flexible solar panels and micro-accelerations on the order of 10^−2^ g. For the spacecraft studied in this work, operation of the optical payload can be paused during the transition from shadow to sunlight and vice versa, so as to avoid the degradation of pointing accuracy induced by thermal shock. Hence, this paper focuses on the suppression of internal micro-vibrations from flywheels and pumps. These disturbances feature small amplitude (μ-g or μrad level), broadband spectrum (0.1 Hz–1 kHz), and quasi-periodic or random time-domain characteristics, which have been verified by on-orbit measurements [8,9,10]. To attenuate micro-vibration disturbances to optical payloads, researchers have conducted comprehensive studies covering disturbance source analysis, micro-vibration suppression instruments, fast steering mirror (FSM)-based high-precision pointing techniques and so on [11,12,13]. Disturbances induced by moving components serve as the primary source of on-orbit micro-vibration for spacecraft. Consequently, numerous researchers have conducted in-depth investigations on this issue [14,15,16,17,18,19]. Masterson et al. [17] developed an empirical harmonic model for reaction wheel disturbances and a MATLAB R2022a toolbox to identify model parameters from test data, showing that the model well predicts frequency characteristics but underestimates disturbances amplified by wheel structural modes. Kim [18] developed a coupled micro-vibration model of reaction wheels and a parameter estimation method, accounting for axial dynamics and measurement offset effects to improve disturbance prediction. Alkomy and Shan [19] established a nonlinear 5-DOF analytical model for reaction wheel micro-vibrations by considering imbalances and bearing waviness. Furthermore, experiments were conducted to verify its effectiveness. Yang et al. [14] proposed a novel disturbance model and identification method for reaction wheel assemblies, which accounted for speed-dependent variable modal frequencies, achieving higher accuracy than conventional empirical and analytical models.

Vibration isolation instruments can effectively attenuate the influence of disturbances on sensitive payloads. They can be installed either between moving components and the spacecraft’s main body or between the spacecraft and sensitive payloads. Kawak [20] proposed a two-stage passive viscoelastic isolation system for control moment gyroscopes that effectively suppressed micro-vibrations while achieving high moment and low power consumption. Lee et al. [21] developed a hybrid vibration isolation system integrating passive three-parameter isolation and active notch filtering. Applied to a multi-axis Stewart platform, it effectively suppressed resonant peaks and achieved high roll-off rates, with tests validating its performance in attenuating satellite micro-vibrations. Chi et al. [22] designed a 6-DOF hybrid vibration isolation platform. By adopting linear active disturbance rejection control, the system eliminates structural resonances, achieves wide-bandwidth isolation, and exhibits strong robustness against modeling uncertainties. Chen et al. [23] proposed a dual passive micro-vibration isolation system using viscoelastic materials for optical satellite flywheels. Ground imaging tests show that the system significantly reduces camera pixel offset, with a maximum RMS attenuation rate of 69.0%.

As the final suppression stage, payload-end line-of-sight (LOS) stabilization and image compensation have become critical for residual vibration mitigation [2]. Fast steering mirrors (FSMs) enable real-time LOS jitter compensation via high-bandwidth actuation. Wu et al. [24] proposed a dual-stage line-of-sight stabilization system consisting of a cubic Stewart platform and a piezoelectric mirror. A hybrid disturbance feedforward method based on gyro and CCD measurements was adopted to broaden the control bandwidth, achieving full-band micro-vibration suppression and high-precision pointing. Li et al., [25] developed a two-stage image stabilization system using bipod vibration isolation legs and a decoupled fast steering mirror (FSM). Experiments showed the system reduced the line-of-sight jitter RMS from 1.253 μrad to 0.276 μrad, achieving sub-microradian stabilization. Sanfedino et al. [26] developed a parametrically uncertain model for large, flexible space structures via the multi-body framework. A robust hybrid control architecture integrating a fast steering mirror and proof-mass actuators was presented to suppress micro-vibrations from reaction wheels and solar array drive mechanisms, realizing high-precision and robust line-of-sight stabilization. Ri et al. [27] proposed a novel two-dimensional vibration isolation system with three quasi-zero stiffness units to suppress vibrations in the plane normal to the LOS. The analysis showed that the system using QZS reduced the vibration by 98.89% in the X-direction and 91.54% in the Y-direction compared to the vibration isolation system with linear springs. Chen et al. [28] presented a dynamic opto-mechanical integrated modeling method combining state-space theory and opto-mechanical coupled ray tracing, enabling efficient and accurate quantitative evaluation of on-orbit imaging quality of space telescopes under micro-vibration disturbances.

A critical outstanding challenge lies in systematically integrating, matching, and collaboratively optimizing these advanced source-isolation and interface-isolation schemes for sensitive optical payloads. An end-to-end integrated suppression chain covering the full path from disturbance sources to optical payload performance needs to be constructed. Moreover, validation in ground tests that approximate realistic on-orbit coupled working conditions still needs to be sufficiently implemented. This challenge serves as the core motivation of the present study.

## 2. Model Development

**Figure 1:**
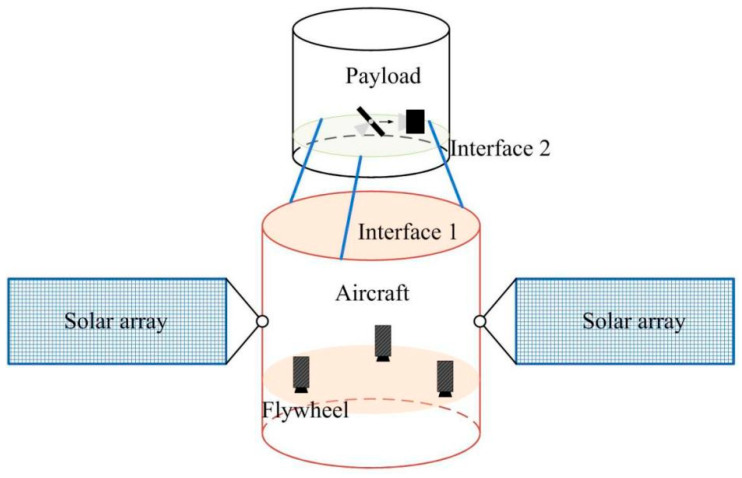
Schematic diagram of the entire spacecraft.

In this paper, a spacecraft carrying sensitive optical payloads, as illustrated in Figure 1, is studied. During on-orbit operation, a spacecraft equipped with sensitive optical payloads identifies a target star in accordance with mission commands and computes the required orbital. The spacecraft then actuates thrusters, reaction wheels, vibration-isolation pointing platforms, and other actuators to steer the target star into the detection field of view of the optical payload, which constitutes the coarse tracking phase. Once entering the capture range, the optical payload images the target star, extracts its actual position via centroid detection, and compares it with the theoretical pointing position to generate a closed-loop error signal. This signal is then used to drive the fast-steering mirror (FSM) system for fine adjustment, thereby stabilizing the target star at the specified position within the field of view (fine tracking).

Micro-vibrations considerably impair the pointing performance of high-performance spacecraft. In particular, unexpected jitter and dynamic responses in sensitive optical payloads are introduced. Focusing on a spacecraft with sensitive optical payloads, a coupled dynamics and control model that integrates micro-vibration disturbance sources, flexible appendages, the main platform structure, vibration-isolation pointing mechanisms, and optical payloads is developed. The proposed model enables full-link prediction and assessment of disturbance identification, micro-vibration suppression, and high-precision pointing.

### 2.1. Disturbance Identification

#### 2.1.1. Flywheel

In the 1980s, NASA analyzed the disturbance forces generated by flywheels and proposed that the disturbance forces and disturbance moments were composed of a series of harmonic functions, as follows:

(1) $$F(t)=\sum_{i=1}^{n}C_{i}f^{2}\sin\left(2\pi h_{i}ft+\alpha_{i}\right)$$

In the formula, *F*(*t*) denotes the disturbance force or disturbance moment, *C_i_* is the amplitude coefficient of the *i*-th harmonic function, *f* represents the rotational frequency of the reaction wheel, *h_i_* stands for the harmonic order, i.e., the ratio of the resonant frequency to the rotational frequency of the reaction wheel, $\alpha_{i}$ is the random phase. In this model, *C_i_* and *h_i_* are the parameters to be identified.

The aforementioned empirical model only captures the speed-dependent frequency characteristics of the flywheel, yet fails to characterize its inherent structural properties. Disturbances originate from static and dynamic imbalances, and the resulting disturbance forces and moments are transmitted to the mounting interface through bearings, support bases, housings, and any available vibration-damping devices. Considering all structures between the flywheel’s rotating shaft and the mounting interface as an integrated assembly and assuming linear system behavior, the disturbance force exerted by the flywheel on the mounting interface can be expressed as

(2) $$F(t)=\sum_{i=1}^{n}\sum_{j=1}^{m}\frac{C_{ij}}{\sqrt{{\left(1-\lambda_{j}^{2}\right)}^{2}+{\left(2\zeta_{j}\lambda_{j}\right)}^{2}}}f^{2}\sin\left(2\pi h_{i}ft+\alpha_{i}\right)$$

In the formula, $C_{ij}$ denotes the amplitude coefficient of the *i*-th harmonic component after transmission through the *j*-th structural mode, $\lambda_{j}=f/f_{j}$ is the frequency ratio of the *j*-th mode, and $\zeta_{j}$ represents the damping ratio of the *j*-th mode.

#### 2.1.2. Pumps

The pump operates at a constant rotational speed during operation. Given that sampled signals in practical experiments are always discrete, the discrete Fourier transform (DFT) algorithm is employed to obtain the discrete amplitude spectrum and phase spectrum of the time-domain disturbance force signals generated by the pump, as follows

(3) $$Y(k)=\sum_{n=0}^{N-1}y(n)e^{-j2\pi kn/N},\;\;\;(k=0,\cdots ,N-1)$$

In the fomula, y(n) denotes the discrete time-domain signal and Y(k) represents the corresponding DFT result. Let the time interval of the discrete sequence be Δt, the sampling frequency be $F_{s}=1/\Delta t$, the number of sampling points be N, and the sampling duration be $T=N\Delta t=N/F_{s}$. The frequency interval (i.e., sampling resolution) associated with Y(k) is then $\Delta f=F_{s}/N$. The corresponding inverse discrete Fourier transform (iDFT) can be expressed as follows.

(4) $$y(n)=\frac{1}{N}\sum_{k=0}^{N-1}Y(k)e^{i2\pi kn/N}$$

Y(k) satisfies the following conjugate relationship (taking *N* as an even number for illustration).

(5) $$Y(k)=Y^{*}(N-2+k)\;,\;(k=1,\cdots ,\frac{N}{2}-1)$$

Although the same sampling frequency can be used across all tests, the test duration differs for each trial, resulting in different frequency resolutions. As a result, direct envelope operations cannot be conducted on the frequency-domain data from different tests. Therefore, the DFT results must be further converted into power spectral density (PSD) results for envelope analysis. The calculation method for PSD is given below (taking N as an even number for illustration).

(6) $$G_{yy}(k)=2\frac{\Delta t}{N}Y(k)Y^{*}(k)=2\frac{\Delta t}{N}{\left|Y(k)\right|}^{2},\;\;\;(k=0,\cdots ,\frac{N}{2})$$

The PSD function characterizes the frequency distribution of vibration energy per unit time and corresponds to the continuous Fourier transform of the autocorrelation function of continuous time-domain signals. According to the definition of “per unit time”, although the above formula presents a discrete form at several frequency points, PSD is essentially a continuous function. Consequently, interpolation can be implemented as required to adjust the frequency resolution. This enables envelope calculation for measured signals with different test durations and frequency resolutions.

### 2.2. Finite Element Modeling

The spacecraft consists of three core subsystems, namely the optical payload, the vehicle platform, and the hybrid active–passive vibration isolation mechanism connecting the above two components. The vehicle platform is integrated with two sets of flexible solar arrays. To achieve effective control and quantitative evaluation of micro-vibration transmission paths, it is necessary to develop a full-spacecraft finite element model that can accurately characterize the dynamic properties and multi-component coupling relationships.

A hierarchical modeling strategy was adopted in the modeling process. First, high-fidelity finite element models of each component were established independently, and then systematic integration was performed through appropriate connection relationships. For the optical payload, the modeling focus lies in the support stiffness characteristics of its internal optical components. Solid elements were employed to simulate the mirror structure, while shell elements were adopted for the supporting truss, and critical points were arranged along the optical axis for micro-vibration assessment. The spacecraft platform was constructed using shell elements, retaining key features such as the primary load-bearing structure and the mounting interfaces for disturbance source equipment (e.g., flywheels and pumps). As typical flexible appendages, the solar arrays were modeled by combining shell elements and spring elements to simulate their deployed and locked state, with full consideration of in-plane, out-of-plane, and torsional vibration modes.

**Figure 2:**
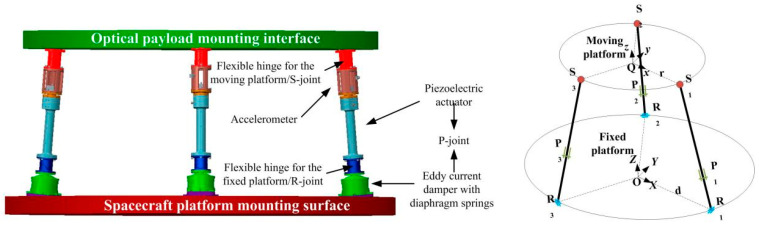
Schematic of the active–passive vibration isolation mechanism.

To reduce the transmission of micro-vibration disturbances from the vehicle platform to the optical payload, a vibration isolation mechanism was installed between the platform and the optical payload. This vibration isolation mechanism adopts a typical 3-SPR parallel mechanism as shown in Figure 2, which consists of an optical payload mounting surface, a spacecraft platform mounting surface, and three legs to form a parallel configuration. Each leg adopts an integrated active–passive vibration isolation design scheme, composed of a piezoelectric actuator, a diaphragm spring, an eddy current damper, an acceleration sensor and two flexure hinges. Among them, the piezoelectric actuator and the diaphragm spring are connected in series to form a prismatic pair (P-joint); the flexure hinge on the optical payload mounting surface adopts a bidirectional grooved design to simulate a spherical pair (S-joint), while the flexure hinge on the spacecraft platform mounting surface adopts a unidirectional grooved design to simulate a revolute pair (R-joint). The three legs are evenly arranged at a circumferential interval of 120°.

**Figure 3:**
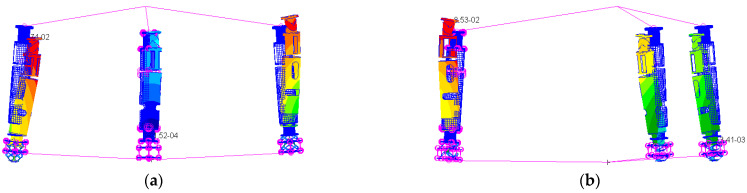
Mode shapes for the active–passive vibration isolation mechanism. (**a**) The first mode shape. (**b**) The second mode shape.

According to the design parameters of the active–passive vibration isolation mechanism, the finite element model was established. Diaphragm springs were modeled using shell elements, the eddy current damper was represented by a spring-damper model, flexure hinges were established with solid elements, and the piezoelectric actuators were implemented as force actuation elements. The first two mode shapes under the fixed constraint of the spacecraft platform are shown in Figure 3, and the first six-order modal frequencies are summarized in Table 1. In Figure 3, yellow denotes minor deformation and red stands for major deformation.

**Figure 4:**
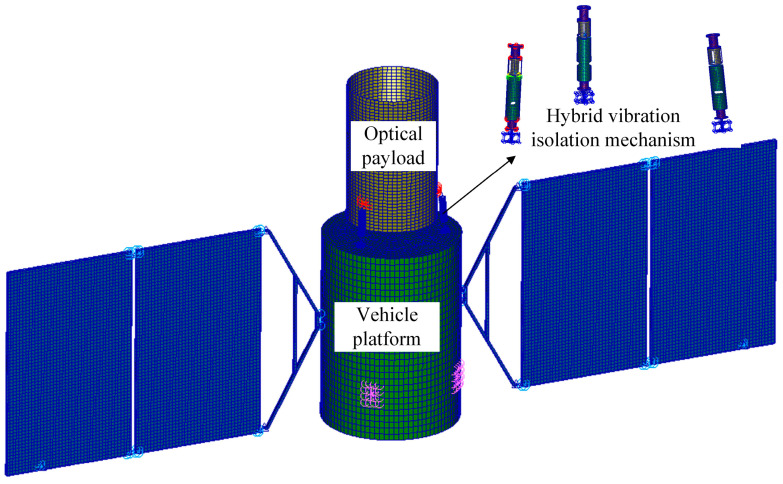
Finite element model of the spacecraft assembly.

By integrating the finite element models of the spacecraft platform, optical payload and active–passive vibration isolation mechanism, the finite element model of the spacecraft assembly was obtained, as shown in Figure 4.

### 2.3. Model Condensation

To improve the computational efficiency and incorporate the active control into the full-spacecraft dynamic model, the Craig–Bampton method was adopted for the dynamic condensation of the global finite element model. The fidelity of the condensed model is directly governed by the selection of interface nodes. In this study, the centroid of the integrated spacecraft was selected as the interface node, which was connected to the primary structure of the spacecraft platform via multi-point constraints. This interface node can characterize the rigid-body motion properties of the entire system. In addition to the interface nodes, a specific number of output nodes with reserved physical degrees of freedom are required.

(1) Disturbance excitation nodes ($m_{1}$): mounting positions of disturbance sources such as flywheels and pumps.

(2) Measurement nodes for active control sensors ($m_{2}$): installation locations of acceleration sensors.

(3) Actuator driving nodes ($m_{3}$): connection points at both ends of the actuators on the vibration isolation mechanism.

(4) Evaluation nodes for the optical payload ($m_{4}$): key positions of optical components.

The undamped governing equation of the full-spacecraft finite element model can be expressed as

(7) $$M\ddot{u}+Ku=F$$

where M is the mass matrix, K is the stiffness matrix, F denotes the external excitation vector, u is the column vector of nodal displacements, and $\ddot{u}$ is the corresponding column vector of nodal accelerations. The six degrees of freedom of the centroid node are arranged in the first six orders of the overall degrees of freedom, and the nodal displacements can therefore be expressed as

(8) $$u=\left[\begin{array}{c} u_{c}\\ u_{i} \end{array}\right]$$

where $u_{c}$ refers to the column vector of centroid node displacements, and $u_{i}$ is the displacement column vector of other nodes. The total dimension of is denoted as N, which represents the total number of degrees of freedom of the model.

According to the two aforementioned equations, the governing equation can be expressed in the partitioned matrix form.

(9) $$\left[\begin{array}{cc} M_{cc} & M_{ci}\\ M_{ci}^{T} & M_{ii} \end{array}\right]\left[\begin{array}{c} {\ddot{u}}_{c}\\ {\ddot{u}}_{i} \end{array}\right]+\left[\begin{array}{cc} K_{cc} & K_{ci}\\ K_{ci}^{T} & K_{ii} \end{array}\right]\left[\begin{array}{c} u_{c}\\ u_{i} \end{array}\right]=F$$

Based on the Craig–Bampton method, the nodal displacement column vector can be approximately expressed as

(10) $$\left[\begin{array}{c} u_{c}\\ u_{i} \end{array}\right]=\left[\begin{array}{cc} I_{6} & 0\\ -K_{ii}^{-1}K_{ci}^{T} & \Phi \end{array}\right]\left[\begin{array}{c} u_{c}\\ \xi \end{array}\right]=T\bar{u}$$

where I is the identity matrix (subscripts indicate matrix dimensions, the same hereinafter), Φ denotes the modal matrix composed of the first $n_{k}$ retained modes, ξ represents the generalized modal coordinates, T is the transformation matrix, and $\bar{u}$ stands for the column vector of all degrees of freedom of the condensed model.

By substituting the above equation into the dynamic governing equation of the full spacecraft and performing left multiplication by $T^{T}$, the condensed governing equation is hence derived.

(11) $$\bar{M}\ddot{\bar{u}}+\bar{K}\bar{u}=\bar{F}$$

where $\bar{M}=T^{T}MT$ is the condensed mass matrix, $\bar{K}=T^{T}KT$ is the condensed stiffness matrix and $\bar{F}=T^{T}F$ is the condensed external excitation.

Meanwhile, to ensure simulation convergence, the damping matrix of the condensed model is defined as follows.

(12) $$\bar{C}=\mathrm{diag}\left(0,0,0,0,0,0,2\eta_{1}\omega_{1},2\eta_{2}\omega_{2},2\eta_{3}\omega_{3},\ldots ,2\eta_{n_{k}}\omega_{n_{k}}\right)$$

where $\eta_{j}$ ($j=1,2,\ldots ,n_{k}$) represents the damping ratio of the *j*-th retained mode.

The damped governing equation after condensation is expressed as

(13) $$\bar{M}\ddot{\bar{u}}+\bar{C}\dot{\bar{u}}+\bar{K}\bar{u}=\bar{F}$$

### 2.4. Control Algorithms

The spacecraft is equipped with multiple control systems. Among these systems, the active vibration suppression control of the active–passive vibration isolation mechanism and the active pointing control of the fast steering mirror dominate the micro-vibration analysis. This section mainly presents the control algorithms of the above two systems.

#### 2.4.1. Control of Vibration Suppression Mechanism

The adopted 3-SPR configuration is a non-redundant parallel mechanism whose number of actuators is equal to the degrees of freedom of the moving platform, yielding a unique inverse kinematic solution. Each attitude or angular motion state of the moving platform is uniquely mapped to the axial elongation or displacement of each leg. In other words, the angular motion of the platform that directly induces optical axis jitter is completely converted into the axial motion of each leg. For the active suppression of angular disturbances, the single-axis acceleration signal measured along the leg axial direction can fully reflect the key micro-vibration characteristics of the moving platform.

**Figure 5:**
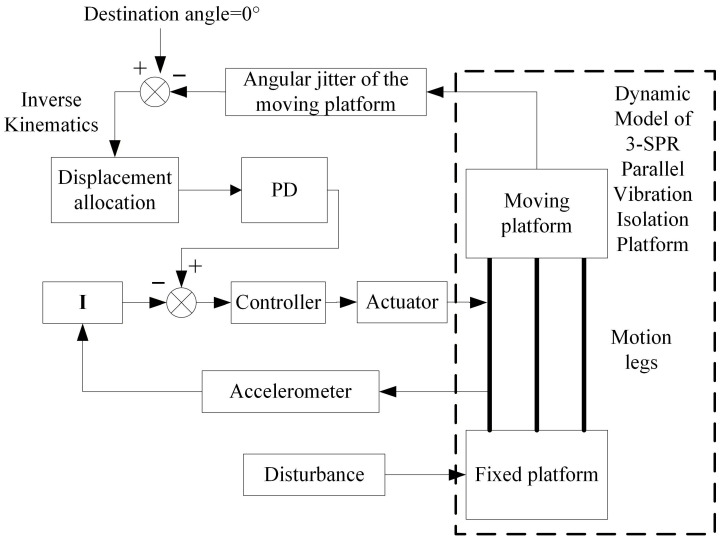
The active control architecture for the 3-SPR vibration isolation mechanism.

The active control architecture for the 3-SPR active–passive vibration isolation mechanism is illustrated in Figure 5. A single-axis accelerometer is mounted at the junction of each motion leg and the moving platform, aligned with the leg’s axial direction to capture the leg’s axial acceleration response. Hence, the integrated acceleration signal is then employed as negative velocity feedback for each leg.

**Figure 6:**
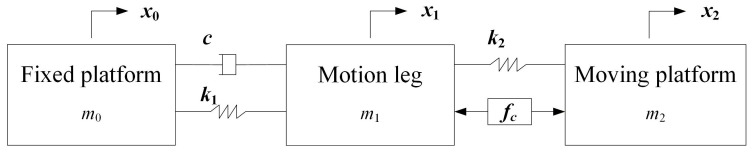
Schematic of a single leg.

The single leg of the active–passive vibration isolation mechanism can be simplified as a three-degree-of-freedom (3-DOF) spring-damping system, as illustrated in Figure 6. In this simplified system, the three mass blocks corresponding to the fixed platform, movable legs and moving platform are denoted by *m*_1_, *m*_2_ and *m*_3_, respectively.

A passive spring-damping isolation system composed of diaphragm springs *k*_1_ and damper *c* is configured between the fixed platform and the movable legs. The movable legs are connected to the moving platform via piezoelectric actuators, whose structural stiffness is *k*_2_. The control forces, *f*_c_, generated by piezoelectric actuators enable active vibration isolation. Denote *x*_0_ as the displacement of the fixed platform (i.e., the external vibration disturbance), *x*_1_ the displacement of the movable leg, and *x*_2_ the displacement of the moving platform. The dynamic governing equations of the system are established as follows.

(14) $$\left[\begin{array}{ccc} m_{0} & 0 & 0\\ 0 & m_{1} & 0\\ 0 & 0 & m_{2} \end{array}\right]\left[\begin{array}{c} {\ddot{x}}_{0}\\ {\ddot{x}}_{1}\\ {\ddot{x}}_{2} \end{array}\right]+\left[\begin{array}{ccc} c & -c & 0\\ -c & c & 0\\ 0 & 0 & 0 \end{array}\right]\left[\begin{array}{c} {\dot{x}}_{0}\\ {\dot{x}}_{1}\\ {\dot{x}}_{2} \end{array}\right]+\left[\begin{array}{ccc} k_{1} & -k_{1} & 0\\ -k_{1} & k_{1}+k_{2} & -k_{2}\\ 0 & -k_{2} & k_{2} \end{array}\right]\left[\begin{array}{c} x_{0}\\ x_{1}\\ x_{2} \end{array}\right]=\left[\begin{array}{c} 0\\ -f_{c}\\ f_{c} \end{array}\right]$$

By applying the Laplace transform to the above equations and considering that the mass of the movable legs is far smaller than that of the fixed and moving platforms, the governing equations involving only the displacements of the fixed platform and moving platform can be given as:

(15) $$\left[m_{2}cs^{3}\;+{\;m}_{2}(k_{1}\;+{\;k}_{2})s^{2}\;+\;k_{2}\mathrm{cs}\;+\;k_{1}k_{2}\right]X_{2}\;=\;\left(k_{2}\mathrm{cs}\;+\;k_{1}k_{2}\right)X_{0}\;+\;\left(\mathrm{cs}\;+\;k_{1}\right)F_{c}$$

An accelerometer is mounted on the moving platform to measure its acceleration response. The measured acceleration signal is integrated to obtain the velocity feedback, which is then utilized to implement closed-loop control via the control force output from the piezoelectric actuators. Furthermore, the leg displacements can be derived from the pose of the moving platform, enabling the introduction of displacement closed-loop control. Considering both the acceleration and displacement closed-loop control loops, the control force can be expressed as follows.

(16) $$f_{c}=-k_{v}{\dot{x}}_{2}\;-\;\left(k_{p}x_{2}+k_{d}{\dot{x}}_{2}\right)$$

Considering both the acceleration closed-loop and displacement closed-loop control, the transfer function from the fixed platform to the moving platform is derived as follows.

(17) $$\frac{X_{2}}{X_{0}}=\frac{k_{2}cs^{2}+k_{1}k_{2}s}{\left\{\begin{array}{r} m_{2}cs^{4}+\left[m_{2}(k_{1}+k_{2})+(k_{v}+k_{d})c\right]s^{3}+\left[(k_{2}+k_{p})c+(k_{v}+k_{d})k_{1}\right]s^{2}+(k_{1}k_{2}+k_{p}k_{1})s \end{array}\right\}}$$

The active control system adopts a digital signal processing scheme. Taking the acceleration signal as an example, the complete link from acquisition to actuation is as follows: single-axis accelerometer → analog-to-digital conversion → on-board digital controller (FPGA) → drive circuit → piezoelectric actuator. The digital architecture is conducive to the realization of complex feedback algorithms, online parameter adjustment and digital coordination with the spacecraft GNC system.

Sensor measurement, analog-to-digital conversion (ADC) and other processes will introduce time delay, which may degrade control accuracy and even affect control stability. In this paper, the influence of time delay is reduced by increasing the control frequency of the control system. The total time of acceleration signal acquisition and ADC conversion is less than 1 ms, ensuring a data sampling frequency higher than 1 kHz. Furthermore, a piezoelectric actuator with a bandwidth exceeding 1 kHz is adopted. Based on the high-sampling-frequency accelerometer and high-bandwidth piezoelectric actuator, the control frequency of the entire system reaches 1000 Hz, meaning that the time from signal acquisition to force output of the piezoelectric actuator is 1 ms. The frequency band targeted for active vibration control in this study ranges from 0.1 Hz to 50 Hz, because vibrations above 50 Hz have been effectively suppressed by the passive isolation system. In addition, the acceleration disturbances above 50 Hz have a negligible effect on the angular disturbance of the optical axis after double integration. Considering that the control frequency of 1000 Hz is much higher than the controlled frequency of 50 Hz, the influence of time delay on control accuracy is very small.

#### 2.4.2. Control of Fast Steering Mirror

**Figure 7:**
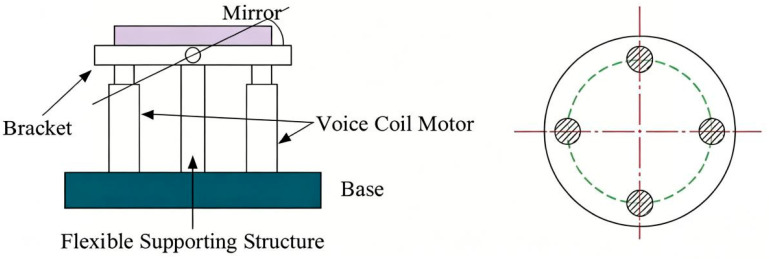
Mechanical structure schematic of the FSM.

In high-precision pointing systems for spacecraft equipped with optical payloads, the fast steering mirror (FSM), as shown in Figure 7, serves as the core actuator of the optical payload system. It suppresses residual line-of-sight (LOS) jitter and realizes terminal-level stabilization and targeting, featuring high bandwidth and high precision. Essentially, it is a two-axis fast tilting mirror driven by piezoelectric ceramics or voice coil motors. Through real-time micro angular deflection, it performs microradian-level dynamic correction on beam directions, so as to compensate for optical axis deviation induced by disturbances such as spacecraft vibration and tracking residuals of the platform.

The spacecraft carrying optical payloads in this paper adopts a classic compound-axis control strategy. As the actuation unit of the fine tracking loop, the FSM receives the spot centroid deviation extracted by a high-frame-rate and high-precision CCD camera. The image-based FSM control system is illustrated in the following figure, which consists of a fast steering mirror, an image sensor, a beacon light, a control unit, and drivers. The control system for FMS adopts a dual-loop structure. Sensors mounted on the fast steering mirror provide feedback signals for the inner loop. Depending on the sensor type, multiple state variables of the tilting mirror can be measured, including position, velocity and acceleration. Meanwhile, the feedback signal of the outer loop is obtained from CCD images, which delivers position information of the target.

**Figure 8:**
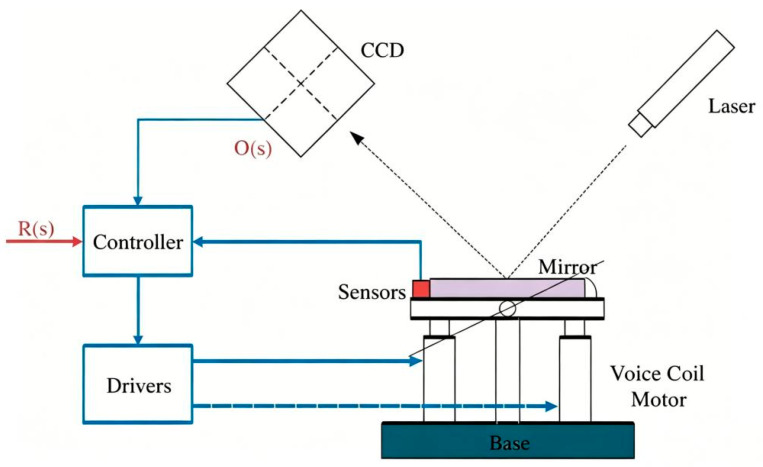
Schematic diagram of the image-based FSM control system.

The corresponding control block diagram is presented in Figure 8. Building upon the coarse tracking system and vibration isolation mechanism, which isolate large-range low-frequency motions and high-frequency disturbances, the core objective of the FSM control system is to further suppress the residual jitter, thereby bringing the LOS pointing accuracy of the optical payload to within the diffraction limit of the optical system. The closed-loop transfer function of the FSM control system is given by

(18) $$\frac{O(s)}{R(s)}=\frac{C(s)G(s)}{1+C(s)G(s)}$$

where *G*(*s*) denotes the plant transfer function, and *C*(*s*) denotes the controller transfer function.

### 2.5. Integrated Modeling

**Figure 9:**
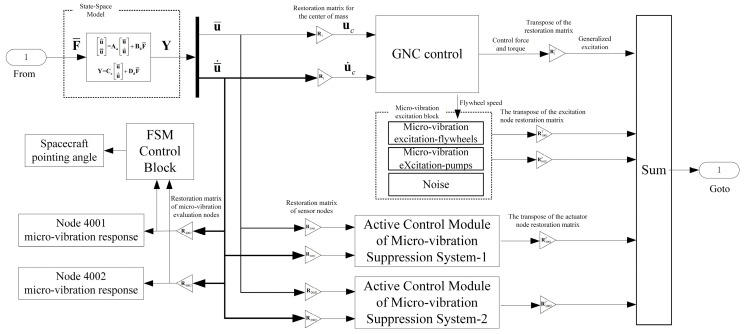
Block diagram of the integrated modeling system.

The integrated modeling of the entire spacecraft was implemented in MATLAB/Simulink. The disturbance forces generated by the flywheels and pumps were calculated using the empirical models identified in Section 2.1. Micro-vibration suppression and FSM control were realized in accordance with the control strategy presented in Section 2.4. The platform GNC control module generated control forces and control moments for coarse pointing, and transmitted real-time flywheel rotational speeds to the flywheel disturbance module. The spacecraft dynamic model established in Section 2 was condensed to obtain the mass, stiffness, and damping matrices using the method shown in Section 2.3. Subsequently, a state-space equation describing the structural characteristics of the spacecraft was formulated based on these matrices. Using this state-space equation, the responses at various positions were calculated according to the disturbance forces and control forces. It should be noted that the forces output by the disturbance modules and control modules are in the physical coordinate system, whereas the inputs and outputs of the state-space equation are in the condensed modal coordinate system. The transformation between the two coordinate systems is achieved through the node restoration matrices. The schematic of the integrated modeling system is shown in Figure 9.

## 3. Results and Discussions

### 3.1. Disturbances

**Figure 10:**
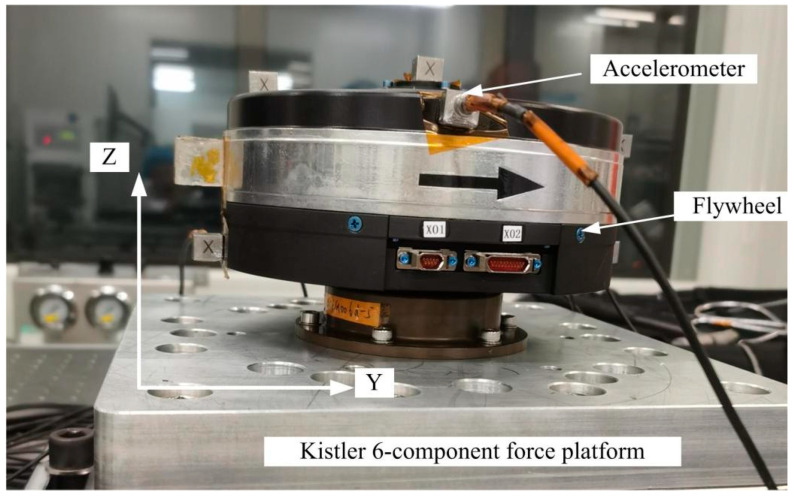
Measuring disturbance forces and moments of the flywheel.

To obtain the disturbance forces and moments of the flywheel, a dynamic force measurement test was conducted on a Kistler 6-component force measuring platform, as shown in Figure 10. The flywheel-induced disturbance forces and moments are closely related to rotational speed and structural characteristics, presenting broadband disturbance features. The flywheel behaves as a time-varying system under variable rotational speed conditions, and its disturbance frequency varies with time.

**Figure 11:**
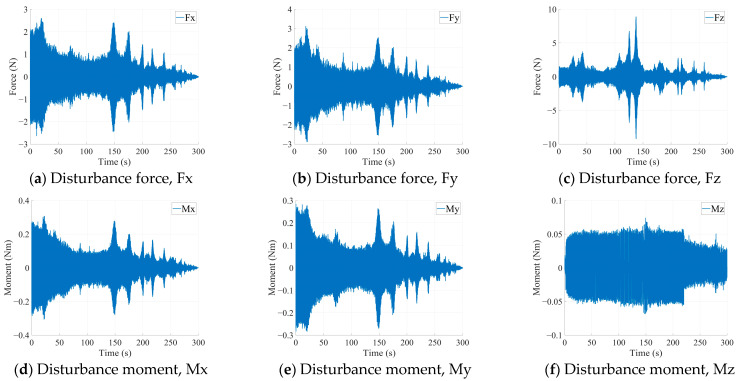
Flywheel disturbance forces and moments in the time domain.

Figure 11 presents the time-domain curves of disturbance forces and moments during the uniform speed reduction of the flywheel from 5000 rpm to zero. It can be observed that, in the time domain, the magnitudes of disturbance forces and moments gradually decrease as the flywheel speed drops from high to low values. Nevertheless, the disturbance force increases at a certain time, such as 150 s, which is caused by the coupling between the flywheel rotational frequency and the structural natural frequency. The disturbance characteristics between *F_x_* and *F_y_*, as well as between *M_x_* and *M_y_*, are highly similar, owing to the central symmetry of the flywheel structure about the *Z*-axis. In particular, the disturbance moment *M_z_* around the *Z*-axis is relatively small without obvious time-frequency characteristics, since the *Z*-axis serves as the main moment output axis of the flywheel.

**Figure 12:**
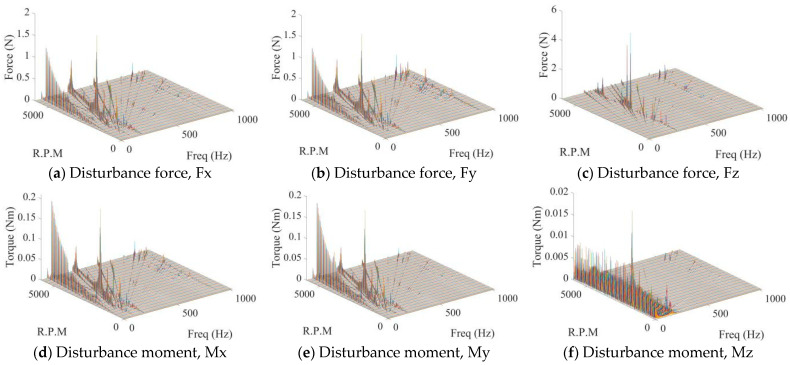
Flywheel disturbance forces and moments in the time-frequency domain.

In variable-speed operating conditions, flywheel disturbances exhibit time-varying characteristics. Conventional Fourier transform is therefore inapplicable, and time-frequency analysis methods such as the short-time Fourier transform are required instead. Figure 12 illustrates the time-frequency characteristics of disturbance forces and moments obtained via the short-time Fourier transform. R.P.M in the figure denotes the rotational speed of the flywheel. Since the flywheel speed varies uniformly with time, the rotational speed axis is equivalent to the time axis. It can be seen that the flywheel disturbances appear as rays passing through the origin in the rotational speed-frequency plane, which characterizes the harmonic behavior of the flywheel and can be characterized by the corresponding term $C_{ij}f^{2}\sin\left(2\pi h_{i}ft+\alpha_{i}\right)$ in Equation (2). In addition, the disturbance forces and moments are significantly amplified at several fixed frequencies, such as 75 Hz and 120 Hz, which are induced by the natural frequencies of the flywheel structure. The coupling effect between disturbances and structural vibration can be represented by the relevant term $1/\sqrt{{\left(1-\lambda_{j}^{2}\right)}^{2}+{\left(2\zeta_{j}\lambda_{j}\right)}^{2}}$ in Equation (2).

**Figure 13:**
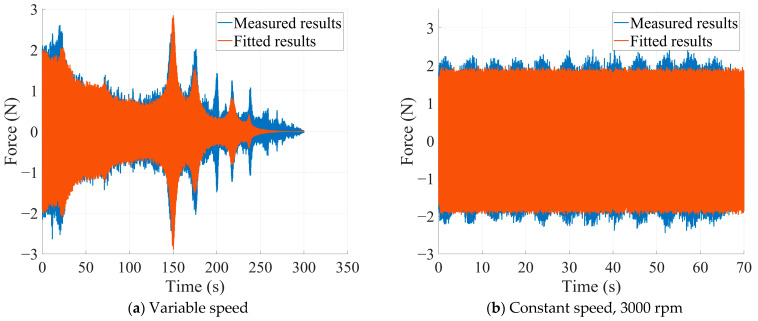
The comparison between the fitted results and the measured results for the flywheel disturbance force.

The comparison between the fitted results based on Equation (2) and the measured results is presented in Figure 13. It can be seen that the fitted data agree well with the measured data under both variable-speed and constant-speed operating conditions. The flywheel disturbance forces and moments obtained by fitting can effectively reflect the amplitude and time-frequency characteristics of the actual flywheel disturbance.

**Figure 14:**
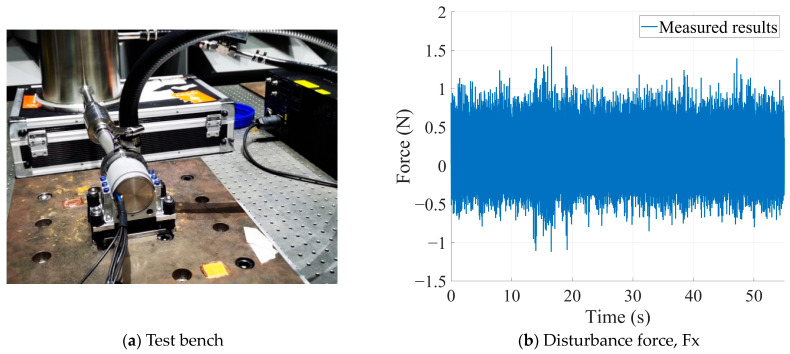
Test bench for disturbances of pumps and the measured disturbance force.

**Figure 15:**
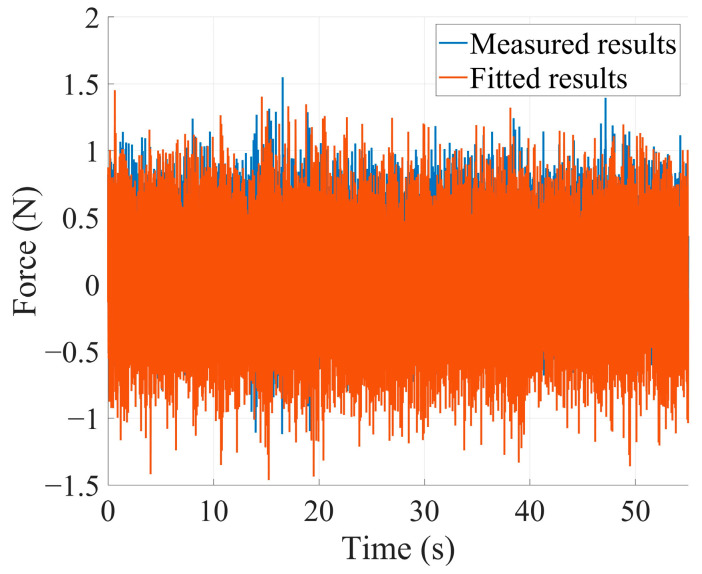
The comparison between the fitted results and the measured results for the pump disturbance force.

Figure 14 and Figure 15 present the measured pump disturbance forces and the comparison between the fitted and measured results. It is indicated that the fitted results are in good agreement with the measured data, which demonstrates that the fitting model can accurately characterize the actual characteristics of pump disturbance forces.

### 3.2. Interface Acceleration

The accelerations at the interfaces of the spacecraft and payload can characterize the micro-vibration environment of the vehicle and serve as a critical basis for evaluating the performance of vibration reduction devices. This section focuses on analyzing the influences of flywheels and pumps on spacecraft interface accelerations under different operating conditions. The division of the spacecraft interfaces is illustrated in the following figure. Interface 1 is the mounting surface between the spacecraft platform and the vibration isolation mechanism, and Interface 2 is the mounting surface between the vibration isolation mechanism and the optical payload.

**Figure 16:**
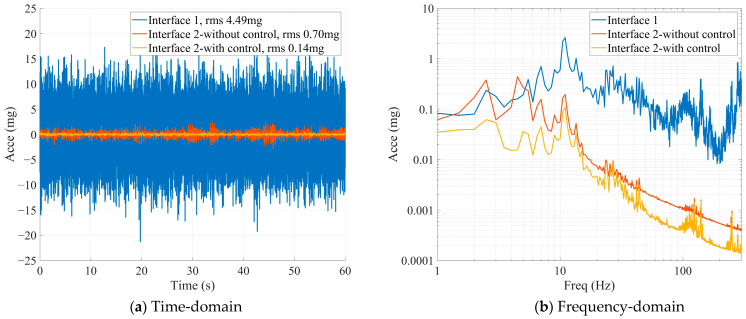
The accelerations of the two interfaces under pump-only operating conditions.

First, the influences of two typical disturbance sources, namely flywheels and pumps, on interface accelerations are analyzed. Figure 16 presents the accelerations of the two interfaces under pump-only operating conditions and compares the performance with and without active control. According to the time-domain results, the variation of the Interface 1 acceleration induced by pump operation is consistent with the pump disturbance force, exhibiting a nearly constant amplitude over time. The peak amplitude is approximately 20.0 mg, with an RMS value of 4.49 mg. Benefiting from the passive vibration isolation of the isolation system, the acceleration is effectively reduced, and the RMS value decreases from 4.49 mg to 0.70 mg. With active control enabled, the acceleration is further suppressed to 0.14 mg. The vibration attenuation effect of passive isolation is approximately −16 dB, while the hybrid active–passive isolation achieves a vibration reduction of about −30 dB. Frequency-domain results further demonstrate the suppression performance of the hybrid active–passive isolation mechanism on acceleration responses within different frequency bands. Distinct acceleration peaks of Interface 1 appear at 12 Hz, 125 Hz and 250 Hz. Specifically, 12 Hz corresponds to the first-order modal frequency of the whole spacecraft, 250 Hz is the rotational frequency of the pump at 15,000 rpm, and 125 Hz denotes the half frequency of 250 Hz. Passive isolation effectively attenuates the vibration peaks at the above three frequency components. Nevertheless, new vibration peaks at 1.8 Hz and 3.5 Hz are introduced during high-frequency vibration attenuation, which correspond to the first-order lateral frequency and first-order rotational frequency of the vibration isolation mechanism, respectively. On the basis of passive isolation, the active control strategy significantly suppresses the response peaks at 1.8 Hz and 3.5 Hz.

**Figure 17:**
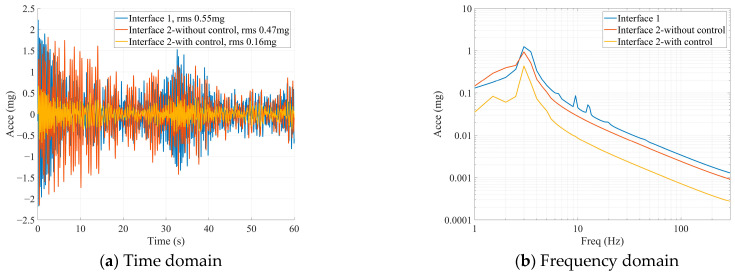
The accelerations of the two interfaces under flywheel-only operating conditions.

Figure 17 shows the acceleration responses at the two interfaces when the flywheel operates alone. The time-domain results indicate that the variation trend of the interface acceleration responses is consistent with that of the flywheel disturbance forces. Their amplitudes vary significantly over time, presenting obvious time-varying characteristics. In addition, the acceleration responses induced by the flywheel are remarkably lower than those caused by the pump. The flywheel-induced acceleration at Interface 1 is only 0.55 mg, whereas the pump-induced acceleration reaches 4.49 mg. According to the disturbance source analysis in Section 3.1, the peak disturbance forces of the pump and flywheel are comparable. However, the flywheel disturbance force is time-varying, with both frequency and amplitude changing dynamically. Its vibration energy is dispersed in the frequency domain, making it difficult to excite structural vibration at specific frequencies. As illustrated by the frequency-domain results, the high-frequency acceleration at Interface 1 excited by the flywheel is rather weak. Since the mid-and high-frequency acceleration components at Interface 1 are inherently low, the vibration suppression performance of the isolation mechanism is less prominent compared with the pump-only operating condition. The attenuation of passive isolation is approximately −1.4 dB, and the overall attenuation of active–passive isolation is about −10.7 dB.

**Figure 18:**
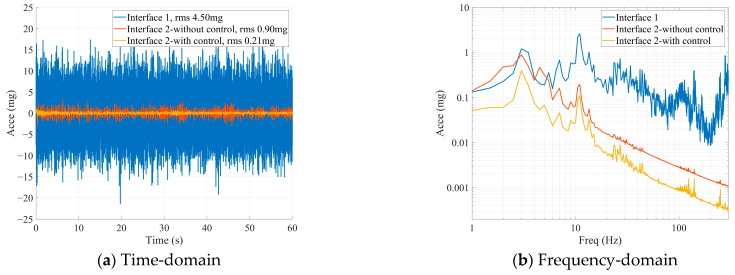
The accelerations of the two interfaces when the pumps and flywheels operate simultaneously.

Figure 18 presents the accelerations at Interface 1 and Interface 2 when the pumps and flywheels operate simultaneously. Since the accelerations induced by the pumps are much larger than those generated by the flywheels, the acceleration responses of both interfaces under the combined operating condition are basically consistent with those in the pump-only working state.

### 3.3. Jitter of LOS

Flywheels and pumps cause micro-vibrations. However, only the angular vibration (pitch or yaw direction) that changes the pointing angle of the optical payload will directly induce LOS jitter and imaging distortion. The translational micro-vibration perpendicular to the optical axis does not change the optical axis pointing, and its impact on the payload performance can be ignored. The 3-SPR hybrid isolation system designed in this paper is mainly aimed at suppressing the angular vibration that affects the optical pointing, which is consistent with the actual mission requirements of high-precision optical payloads. This section focuses on analyzing the influences of pumps and flywheels on the jitter of LOS.

**Figure 19:**
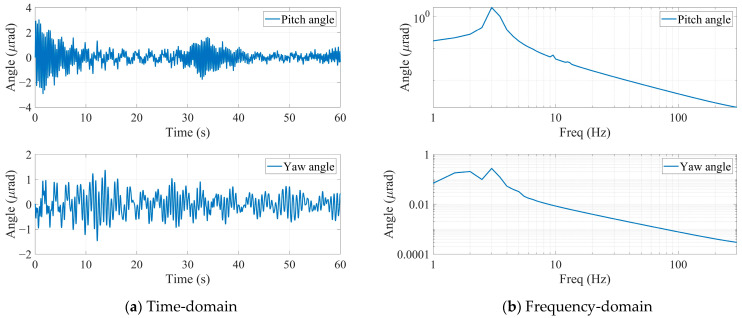
Pitch and yaw angles of the LOS under flywheel-only operation.

**Figure 20:**
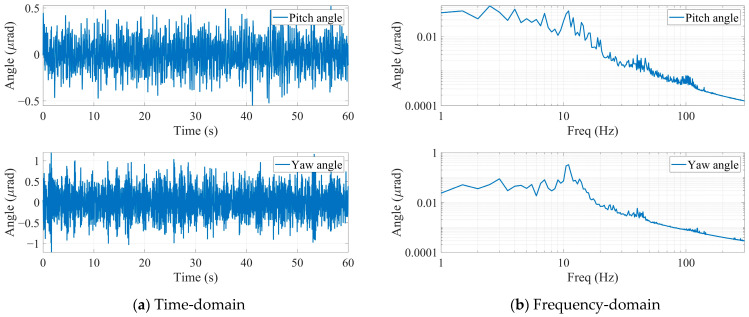
Pitch and yaw angles of the LOS under pump-only operation.

Figure 19 and Figure 20 illustrate the variations in the pitch and yaw angles of the optical axis under pump-only and flywheel-only operating conditions, respectively. It can be observed that the jitter of LOS induced by the flywheel yields RMS values of 0.63 μrad for the pitch angle and 0.55 μrad for the yaw angle. In comparison, the RMS values of the pitch and yaw angles caused by the pump are 0.21 μrad and 0.31 μrad. The jitter of LOS induced by the flywheel is greater than that induced by the pump, which is contrary to the influence law of flywheels and pumps on acceleration analyzed in Section 3.2. This is mainly attributed to the fact that the acceleration disturbance excited by the pump is concentrated in the medium- and high-frequency bands. After two integrations, the contribution of medium- and high-frequency acceleration disturbances to angular jitter becomes negligible.

**Figure 21:**
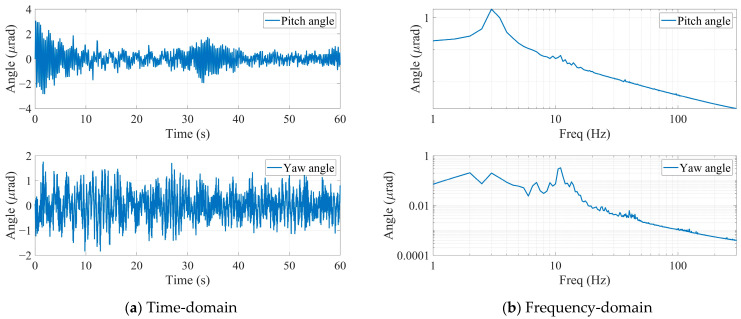
Pitch and yaw angles of the LOS when the flywheels and pumps operate simultaneously.

Figure 21 shows the variations in the pitch and yaw angles of the LOS when the flywheels and pumps operate simultaneously. The RMS value of the pitch angle is 0.66 μrad, and that of the yaw angle is 0.63 μrad. In terms of time-domain waveforms, the time-domain curves of the pitch and yaw angles under combined operation approximate the superposition of the responses generated by the flywheel and pump individually. In terms of RMS values, the pitch angle satisfies 0.66 μrad $\approx \sqrt{0.63^{2}+0.21^{2}}\;$μrad, and the yaw angle satisfies 0.63 μrad $\approx \sqrt{0.55^{2}+0.31^{2}}\;$μrad.

### 3.4. Test Verification

**Figure 22:**
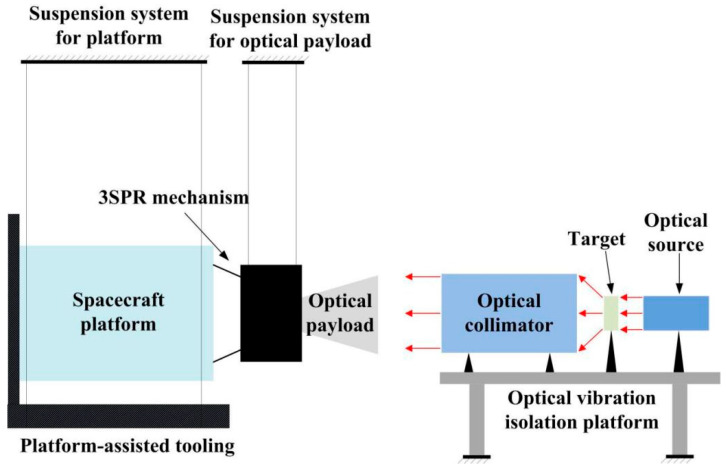
Schematic of whole-spacecraft micro-vibration test.

The overall experimental layout is illustrated in Figure 22. The spacecraft platform and optical payload are horizontally gravity-unloaded via a suspension system. A collimator, target and light source are arranged directly in front of the payload. The light source passes through the target to provide point target information. After simulating an infinite-distance target through the collimator, the target light enters the field of view of the optical payload. The image centroid is subsequently extracted to obtain the image shift variation, thereby realizing direct measurement of the pitch and yaw pointing accuracy induced by micro-vibration.

**Figure 23:**
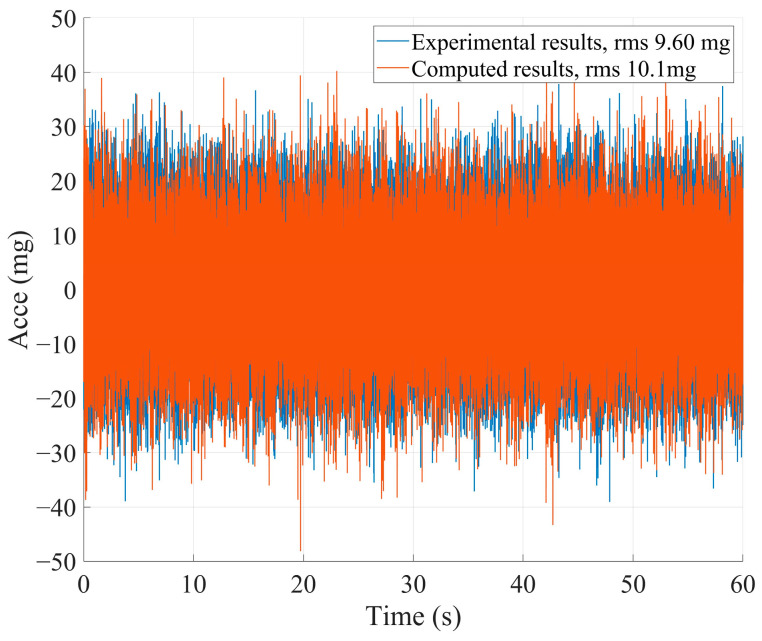
Comparison of accelerations at Interface 1.

**Figure 24:**
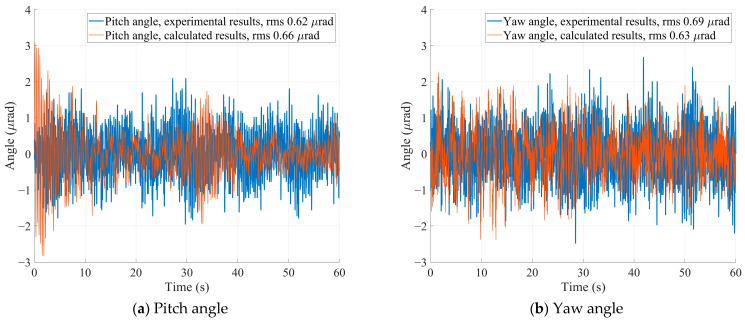
Comparison of pitch and yaw angles of LOS.

With the spacecraft platform and optical payload kept in a suspended state, micro-vibration disturbance sources, including flywheels and pumps, are activated. Accelerometers are used to measure the acceleration at each interface, and the CCD camera integrated in the optical payload is adopted to acquire the angle of LOS. The experimental data are further compared with the model-based calculation results. Figure 23 and Figure 24 present the comparison of the acceleration at Interface 1 and the optical axis pointing angle, respectively. It can be observed that the time-domain variation trends and RMS values of the experimentally measured accelerations are in good agreement with the calculated results, which verifies the accuracy of the acceleration prediction. Moreover, the calculated pitch and yaw angles also match the experimental data well. The pitch angle is significantly affected by the flywheel, and its time-domain waveform exhibits obvious time-varying characteristics, which are consistently reflected in both calculated and measured results. By contrast, the yaw angle is dominated by the pump disturbance with a weak time-varying amplitude. In terms of RMS errors, the relative deviation of the pitch angle between simulation and experiment is approximately 6.5%, while that of the yaw angle is about −8.7%. Both the acceleration and LOS angular results demonstrate the validity and accuracy of the analytical model established in this paper.

## 4. Conclusions

This paper develops a full-link integrated dynamic model of a spacecraft that couples disturbance sources, flexible structures, hybrid active–passive vibration isolation and high-precision pointing control. Furthermore, a systematic ground test verification is performed. The main conclusions are as follows:

1. A refined disturbance model is established for flywheels and pumps. The flywheel disturbance presents obvious time-varying characteristics under variable speed, while the pump disturbance is concentrated in medium and high frequency bands, which is consistent with test measurements.
2. The hybrid active–passive vibration isolation mechanism exhibits excellent suppression effect on micro-vibration induced by flywheels and pumps. Passive isolation reduces the acceleration by -16 dB, and active control further improves the attenuation to -30 dB, effectively suppressing the resonant peaks introduced by passive isolation.
3. The influence of the laws of flywheels and pumps on interface acceleration and line-of-sight jitter is opposite. The pump causes large acceleration but small angular jitters due to the attenuation of medium-high frequency components after double integration, while the flywheel induces small acceleration but remarkable time-varying angular jitter.
4. The integrated model is validated by the whole-spacecraft test. The calculated results of accelerations and optical axis pointing angles are in good agreement with the test results, verifying the accuracy and effectiveness of the proposed modeling method.

The proposed full-link analysis and verification framework provides a feasible technical approach for micro-vibration prediction and suppression of high-precision optical spacecraft disturbed by rotating components such as flywheels and pumps. For external disturbances such as thermal shock, subsequent research will focus on integrating them into the integrated dynamic model to realize a more comprehensive disturbance suppression analysis.

## Figures and Tables

**Table 1:** Comparison of finite element analysis and modal test results.

Order	Finite Element Analysis (Hz)	Mode Test (Hz)	Description
1	2.15	2.11	Translation along the *X*-axis
2	2.16	2.18	Translation along the *Y*-axis
3	3.03	2.93	Rotation about the *X*-axis
4	3.05	2.96	Rotation about the *Y*-axis
5	3.51	3.35	Rotation about the *Z*-axis
6	4.12	4.02	Translation along the *Z*-axis

## Data Availability

The original contributions presented in this study are included in the article. Further inquiries can be directed to the corresponding author.
